# Immunological correlates of mycobacterial growth inhibition describe a spectrum of tuberculosis infection

**DOI:** 10.1038/s41598-018-32755-x

**Published:** 2018-09-27

**Authors:** Matthew K. O’Shea, Rachel Tanner, Julius Müller, Stephanie A. Harris, Danny Wright, Lisa Stockdale, Elena Stylianou, Iman Satti, Steven G. Smith, James Dunbar, Thomas E. Fletcher, Martin Dedicoat, Adam F. Cunningham, Helen McShane

**Affiliations:** 1 The Jenner Institute, Nuffield Department of Medicine, University of Oxford, Oxford, UK; 2 Institute of Microbiology and Infection, University of Birmingham, Birmingham, UK; 3 Department of Immunology and Infection, London School of Hygiene and Tropical Medicine, London, UK; 4 The Friarage Hospital, Northallerton, Yorkshire UK; 5 Liverpool School of Tropical Medicine and Hygiene, Liverpool, UK; 6 Heartlands Hospital, Birmingham, UK; 7 Institute of Immunology and Immunotherapy, University of Birmingham, Birmingham, UK

## Abstract

A major contribution to the burden of Tuberculosis (TB) comes from latent *Mycobacterium tuberculosis* infections (LTBI) becoming clinically active. TB and LTBI probably exist as a spectrum and currently there are no correlates available to identify individuals with LTBI most at risk of developing active disease. We set out to identify immune parameters associated with *ex vivo* mycobacterial growth control among individuals with active TB disease or LTBI to define the spectrum of TB infection. We used a whole blood mycobacterial growth inhibition assay to generate a functional profile of growth control among individuals with TB, LTBI or uninfected controls. We subsequently used a multi-platform approach to identify an immune signature associated with this profile. We show, for the first time, that patients with active disease had the greatest control of mycobacterial growth, whilst there was a continuum of responses among latently infected patients, likely related to the degree of immune activation in response to bacillary load. Control correlated with multiple factors including inflammatory monocytes, activated and atypical memory B cells, IgG1 responses to TB-specific antigens and serum cytokines/chemokines. Our findings offer a method to stratify subclinical TB infections and the future potential to identify individuals most at risk of progressing to active disease and benefit from chemoprophylaxis.

## Introduction

Interrupting tuberculosis (TB) transmission by prompt diagnosis and treatment of active disease has led to substantial reductions in TB prevalence and mortality. However, TB still represents a huge challenge to global health with an estimated 10.4 million new cases and 1.7 million deaths attributed to TB in 2016^1^. Modeling estimates that even if transmission was stopped completely and instantly, reactivation from the 2 billion individuals estimated to have latent *Mycobacterium tuberculosis* (*M*.*tb*) infections (LTBI) would cause >100 cases per million population in 2050^2^. There is increasing recognition that managing individuals with LTBI is needed^3^. However, the estimated lifetime risk of LTBI progressing to disease is only 12% and there are no tests to identify this population^4^. A test to stratify these high-risk individuals within the latent reservoir would facilitate targeted preventative therapy, offering an alternative solution to the economic and logistic challenge of treating the global LTBI population.

Identifying a suitably robust and affordable diagnostic test, capable of distinguishing high and low risk individuals is problematic. Most research has assessed individual immune parameters or techniques to identify correlates of TB risk and protection^5^. Given the complexity of TB immunology and pathogenesis a single measure of immune function may be insufficient to identify risk of LTBI reactivation and therefore a combination approach may be required^6^.

A new approach is needed to how we view the traditional clinical phenotypic description of TB. The binary classification of TB as distinct, homogeneous entities of active disease and LTBI is likely an oversimplification and these entities actually show marked biological heterogeneity, corresponding to partially overlapping regions on a spectrum of *M*.*tb* infection^7,8^. This continuous spectrum of responses to *M*.*tb* probably reflects the contributions of innate and acquired immunity to control, resulting in quiescent infection, active/subclinical disease, and active/fulminant disease^8^.

*Ex vivo* mycobacterial growth inhibition assays (MGIAs) are functional assays that provide a summative assessment of a range of immune mechanisms within a biological sample^9^. In the absence of defined mechanisms, this assay provides an unbiased assessment of mycobacterial control as a measure of anti-mycobacterial immunity.

We hypothesised that individuals with different degrees of *M*.*tb* infection would show differential anti-mycobacterial responses in a MGIA, thus defining a spectrum of infection.

Here, we describe a novel exploratory study using a whole blood MGIA to measure the capacity of individuals with active TB disease, LTBI and uninfected healthy volunteers, to control mycobacterial growth *ex vivo*, before and after TB treatment. Characterisation of MGIA control profiles identified associations with several different immunological and cellular factors. Based on the delineation of the spectrum of *M*.*tb* infection we suggest a profile of active/subclinical disease that may be used in developing predictive models of reactivation.

## Results

### Demographic and haematological characteristics of study participants

Samples were collected from 211 HIV-negative adults, including 21 with active TB disease, 139 with LTBI and 51 uninfected healthy controls (Table 1). Mean age was 27.1 years. Most participants were male (76.3%) and born outside the UK (80.1%). Pulmonary TB was the most common diagnosis in active disease (71.4%) and 38.1% were confirmed microbiologically. Each patient with active disease completed 6 months anti-TB treatment. The majority of individuals with LTBI (94.2%) completed 3 months of rifampicin and isoniazid preventative therapy.

**Table 1:** Characteristics of study participants.

	State of infection			P values		
	Active TB	LTBI	Controls	P1	P2	P3
	n = 21 (%)	n = 139 (%)	n = 51 (%)			
Mean age (yrs) [IQR]	33.4 [22.5–44]	23.7 [19–28.5]	24.1 [19.4–27]	<0.0001	0.0065	0.54
Gender male	16 (76.2)	107 (77.0)	38 (74.5)	0.9966	0.9874	0.9339
**Place of birth**						
UK	9 (42.9)	15 (10.8)	18 (35.3)	0.0008	0.5985	0.0002
Outside UK	12 (57.1)	124 (89.2)	33 (64.7)	—	—	—
**Ethnic group**						
White Caucasian	2 (9.5)	8 (5.8)	18 (35.3)	0.6216	0.0409	0.0001
Sub-Saharan African	4 (19.0)	23 (16.5)	1 (2.0)	0.7580	0.0233	0.0057
Indian subcontinent	13 (61.9)	99 (71.2)	32 (62.7)	0.4452	1.0	0.2906
Other	2 (9.5)	9 (6.5)	0	0.6393	0.0822	0.1159
BCG vaccinated	20 (95.2)	111 (79.9)	31 (60.8)	0.1275	0.0036	0.0135
Culture confirmed TB	8 (38.1)	NA	NA	—	—	—
**System**						
Pulmonary	15 (71.4)	NA	NA	—	—	—
Extrapulmonary^a^	5 (23.8)	NA	NA	—	—	—
Miliary	1 (4.8)	NA	NA	—	—	—
Completed treatment	21 (100)	131 (94.2)^b^	NA	0.5982	—	—
Post-treatment samples	12 (57.1)	78 (56.1)	NA	1.0	—	—

Two-tailed P values were calculated by Fisher’s exact test between infection states as follows: P1 = Active TB vs LTBI; P2 = Active TB vs Healthy controls; P3 = LTBI vs Healthy controls.

^a^Extrapulmonary TB included four lymph node TB and one bone TB.

^b^n = 8 (5.8%) participants with LTBI were untreated due to the following reasons: n = 4 were >35 years of age; n = 3 were pregnant; n = 1 reported drug intolerance shortly after commencing therapy.

LTBI = latent TB infection; IQR = interquartile range; NA = not applicable.

### *Ex vivo* differential mycobacterial growth is associated with disease state

**Figure 1:**
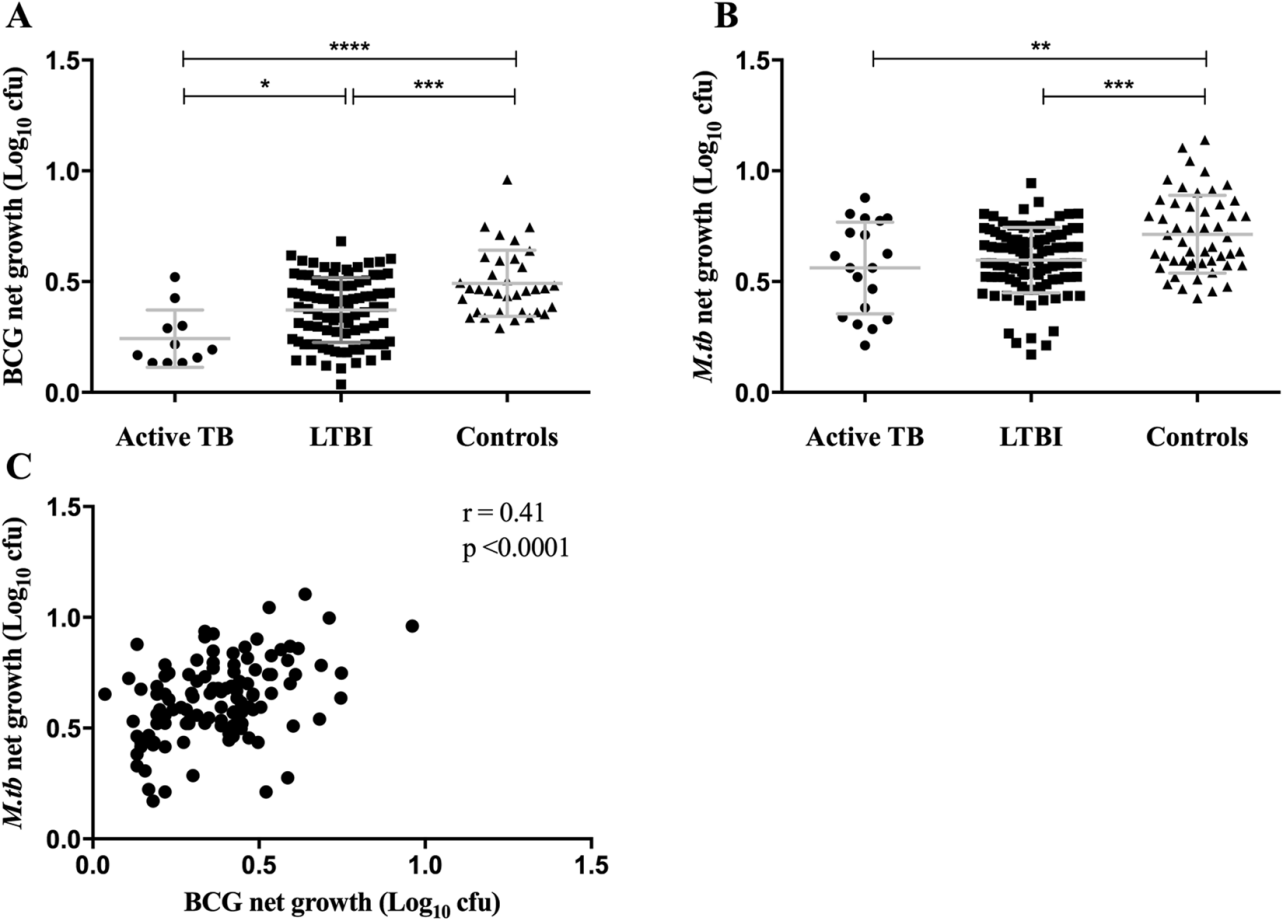
*Ex vivo* differential mycobacterial control is associated with disease state in pre-treatment whole blood. Samples were collected from volunteers before starting anti-TB treatment. Whole blood MGIT using BCG Pasteur (**A**) and *M*.*tb* H37Rv (**B**) was performed and data meeting the inclusion criteria (duplicate ΔTTP < 6 hours) are shown (BCG: n = 11, n = 93, n = 35; H37Rv: n = 19, n = 101, n = 51; for active TB, LTBI and healthy controls, respectively). The association between BCG and H37Rv pre-treatment MGIT results was evaluated by Pearson’s correlation (**C**). Points represent the mean of duplicates; bars represent mean values with SD. A one-way multiple comparison ANOVA with Tukey’s post-test correction was performed between the groups. *Represents a p-value of <0.05, **a p-value of <0.005, ***a p-value of <0.0005 and ****a p-value of <0.0001.

The functional ability of pre-treatment whole blood samples to control mycobacterial growth was evaluated using the MGIA. A total of 139/211 results were available using BCG and 171/211 using *M*.*tb* in the MGIT assay. Reduced mycobacterial growth was seen among individuals with active TB compared to healthy controls (Fig. 1A,B). A wide spectrum of growth rates occurred in the LTBI group but significant differences in net growth between active and LTBI groups was only seen with the BCG MGIT. When BCG was used in this assay, the level of growth observed was lowest for active TB patients, greater growth in samples from latently infected individuals and the greatest growth in healthy controls. In contrast, for the *M*.*tb* MGIT there was similar control of bacterial growth by samples from active TB or latently infected individuals. Net growth was significantly higher with *M*.*tb* than BCG for active, LTBI and control groups (p = 0.01, p < 0.0001, p < 0.0001, respectively). There was a strong correlation between BCG and *M*.*tb* net growth (r = 0.41, p < 0.0001) (Fig. 1C). Thus, greater differential mycobacteria growth occurred in the modified BCG MGIT and was dependent upon the infection status of the subject.

### Mycobacterial growth increases in the whole blood assay following treatment for TB

**Figure 2:**
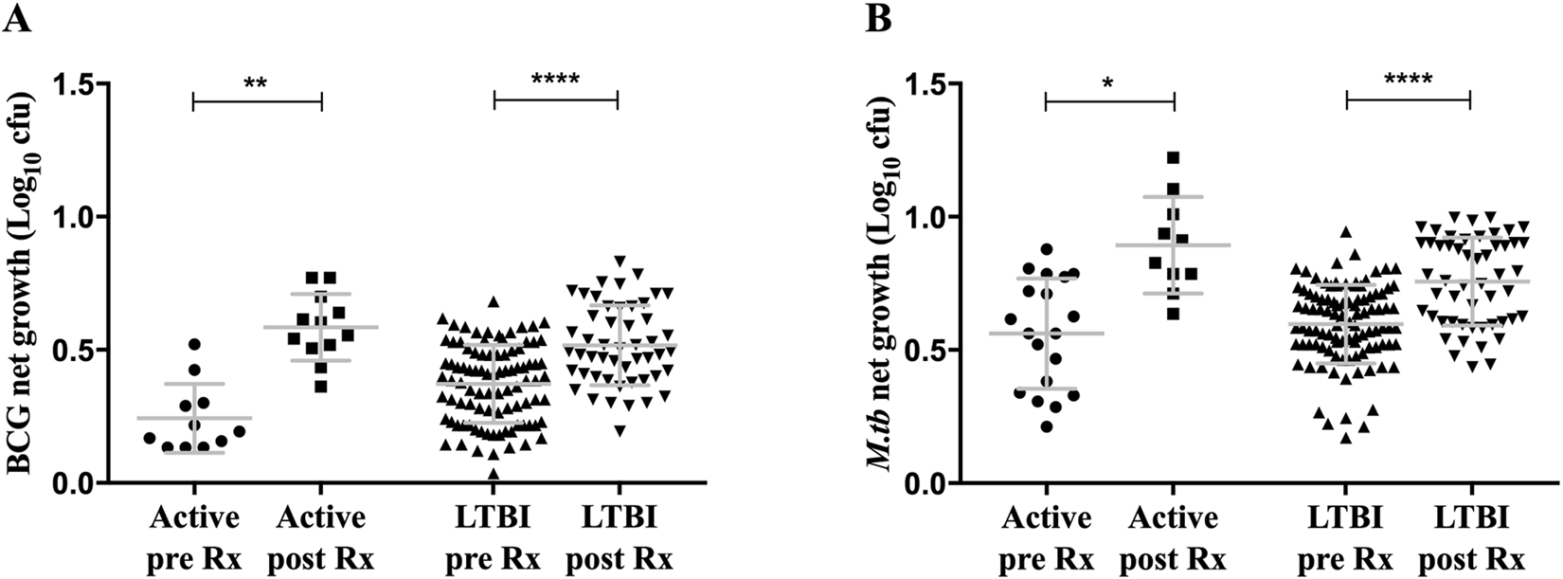
Enhanced mycobacterial control decreases following TB treatment. Samples were collected from volunteers with active TB and LTBI between 1 to 6 months following completion of anti-TB treatment. Whole blood MGIT using BCG Pasteur (**A**) and *M*.*tb* H37Rv (**B**) was performed. Data meeting the inclusion criteria (duplicate ΔTTP < 6 hours) are shown. For MGIT data, points represent the mean of duplicates; bars represent mean values with SD. A paired t- test was performed to assess net growth before and after treatment. *Represents a p-value of <0.05, **a p-value of <0.01 and ****a p-value of <0.0001. Red circles = active TB and black = LTBI.

The MGIT assay was repeated after completion of treatment for active disease and LTBI (n = 62). Significant increases in the growth of both BCG and *M*.*tb* were observed in each group (Fig. 2A,B). The increase in BCG net growth was greatest in blood from the active TB group (mean growth pre- vs post-treatment: active TB, p = 0.006; LTBI, p < 0.0001). Similar changes in the growth of *M*.*tb* following treatment were also seen (mean growth pre- vs post-treatment: active TB, p = 0.031; LTBI, p < 0.0001).

### *Ex vivo* mycobacterial control correlates with haematological parameters

Significant differences in pre-treatment haematological parameters were observed (n = 183) (Supplementary Table 1). Compared to LTBI or healthy controls, individuals with active disease had significantly more monocytes (both p < 0.0001) and fewer lymphocytes (p = 0.017 and p = 0.003, respectively), resulting in a significantly increased monocyte-to-lymphocyte (ML) ratio (both p < 0.0001). Haemoglobin concentrations were significantly reduced in both active disease and LTBI when compared with uninfected controls (p = 0.038 and p = 0.0003, respectively). Following TB treatment, active TB and LTBI patients showed significant reductions in total white cells (p = 0.039 and p < 0.0001), neutrophils (p = 0.008 and P < 0.0001), monocytes (p = 0.004 and p = 0.041) and platelet count (p = 0.026 and p < 0.0001), together with increases in haemoglobin concentration (p = 0.004 and p = 0.014, respectively), compared with pre-treatment levels (n = 89). There was a non-significant trend for increased lymphocytes in active patients and the ML ratio fell significantly after treatment in this group (p = 0.008).

Given that whole blood samples contain several cellular constituents, which may have anti-mycobacterial functions, we examined which factors correlated with the mycobacterial growth rate. For BCG, significant positive correlations were seen between net growth and lymphocyte counts (rho = 0.19, p = 0.038) and haemoglobin concentrations (rho = 0.29, p = 0.002), together with a non-significant negative association between ML ratio and mycobacterial growth (rho = −0.16, p = 0.07) (Supplementary Fig. 1A–C). For *M*.*tb*, monocyte count and ML ratio were significantly negatively correlated (rho = −0.20, p = 0.015; rho = −0.17, p = 0.046, respectively) and haemoglobin concentration and mean cell volume were positively correlated (rho = 0.33, p < 0.0001; rho = 0.23, p = 0.007, respectively) with net growth (Supplementary Fig. 1D–G). In a reversal of the pre-treatment relationships, significant positive correlations between *M*.*tb* net growth and monocyte count (rho = 0.22, p = 0.05) and ML ratio (rho = 0.4, p = 0.0005), together with a negative correlation with lymphocyte count (rho = −0.32, p = 0.006), were observed after treatment (Supplementary Fig. 1H–J).

### Distinct monocyte subsets are associated with differential *ex vivo* mycobacterial control

**Figure 3:**
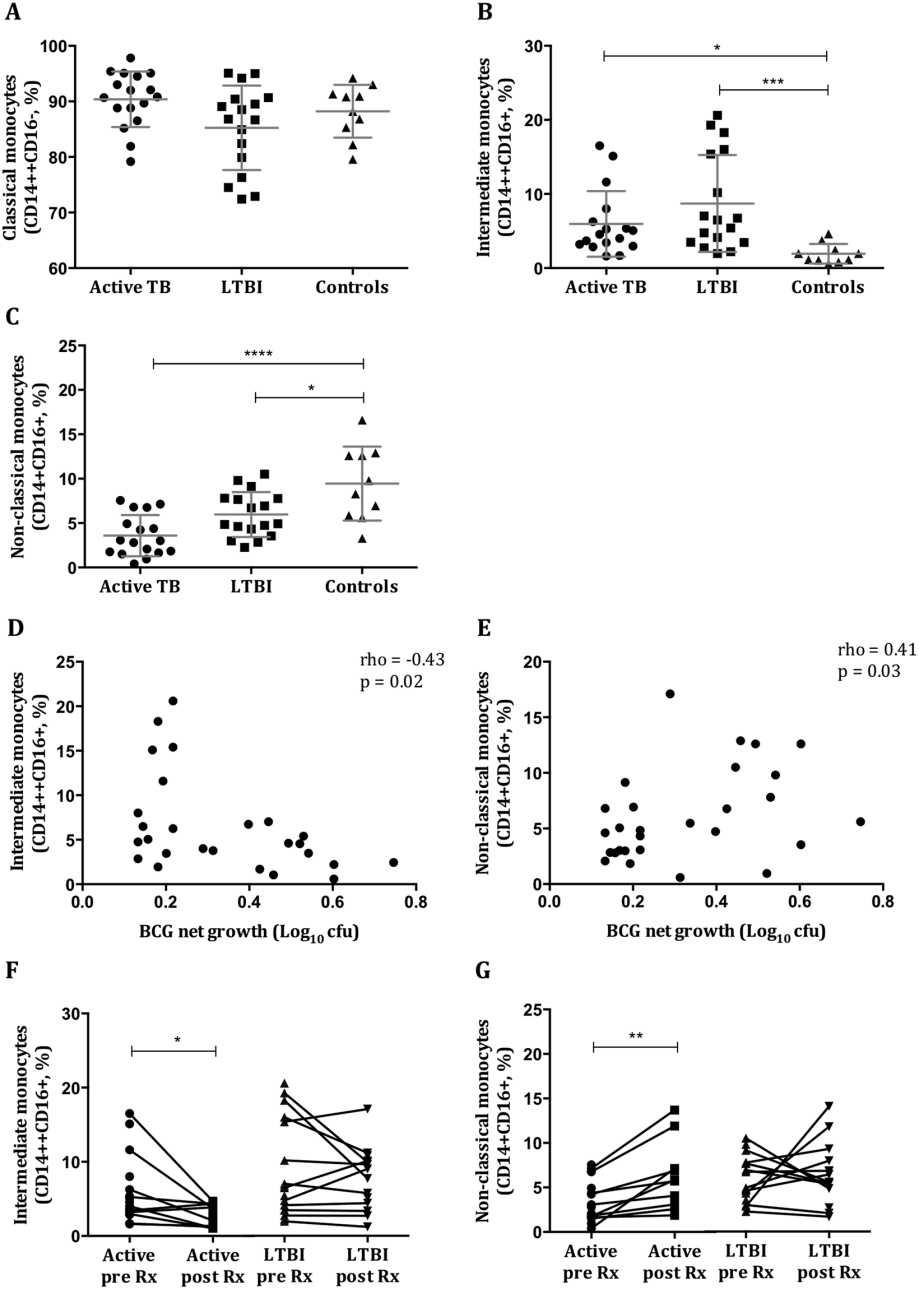
Altered proportions of intermediate and non-classical monocyte subsets are associated with differential mycobacterial control. Monocyte subsets were characterized among patients with active TB disease (n = 17), LTBI (n = 17) and healthy controls (n = 10) before starting anti-TB treatment. The proportions of classical (**A**), intermediate (**B**), and non-classical (**C**) monocytes were calculated from the total monocyte population. A negative correlation between intermediate monocytes and net growth (**D**), and a positive correlation between non-classical monocytes and net growth (**E**) of BCG were seen. The latter was also seen with *M*.*tb* H37Rv (data not shown). Monocyte subsets were then characterized after completion of treatment among active TB (n = 11) and LTBI (n = 14) patients. The proportions of intermediate (**F**) and non-classical (**G**) monocytes are shown. Points are single values and bars represent the mean with SD. After testing for normality, an ordinary one-way ANOVA with Tukey’s correction (**A**,**C**,**G**) or a Kruskal-Wallis test with Dunn’s correction for multiple comparisons (**B**,**F**) was performed. *Represents a p-value of <0.05, **a p-value of <0.005, ***a p-value of <0.0005 and ****a p-value of <0.0001. For correlations Spearman’s rho and associated p-values are shown.

Monocytes play an important role in the immune response to TB and therefore subsets were characterised in pre- (n = 44) and matched post-treatment (n = 22) samples. No differences in the proportion of classical monocytes between TB, LTBI and healthy control groups were noted (mean = 90.4%, 85.3%, 88.2%, respectively) (Fig. 3A). However, significant increases in the proportion of intermediate monocytes in the *M*.*tb*-infected groups compared to healthy controls were seen (mean = 5.9%, 8.7%, 1.9%; active, LTBI, controls, respectively; active vs controls p = 0.012; LTBI vs controls p = 0.0004), together with reductions in non-classical monocytes (mean = 3.6%, 5.9%, 9.4%; active, LTBI, controls, respectively; active vs controls p < 0.0001; LTBI vs controls p = 0.012) (Fig. 3B,C). There was a significant negative correlation between the proportion of intermediate monocytes and BCG net growth by MGIA (rho = −0.43, p = 0.02) (Fig. 3D) and positive correlations between non-classical monocytes and net growth of both BCG (rho = 0.41, p = 0.03, Fig. 3E) and *M*.*tb* (rho = 0.33, p = 0.03, data not shown). Following anti-TB treatment there were significant reductions in the proportion of intermediate monocytes (p = 0.037) and increases in non-classical monocytes (p = 0.004) in active TB patients, resulting in profiles similar to healthy controls, but no changes among LTBI patients (Fig. 3F,G). There were no changes in classical monocytes in any group (data not shown). Thus mycobacterial growth inhibition was associated with more intermediate and fewer non-classical monocytes and altered subset frequencies typically normalised following successful treatment.

### Selective B cell subsets increase in *M*.*tb* infection and correlate with improved control

**Figure 4:**
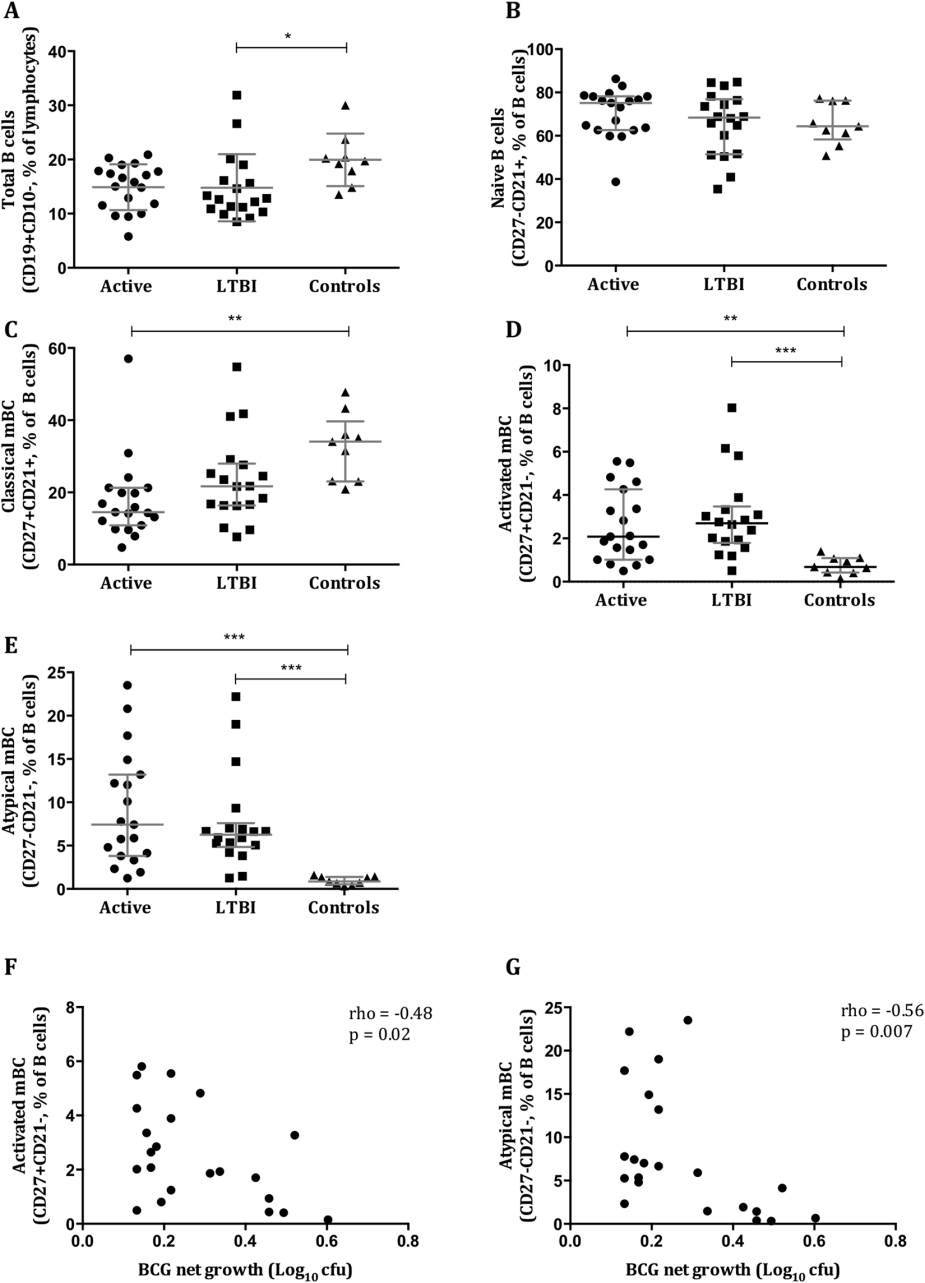
Increases in activated and atypical memory B cells occur in *M*.*tb* infection and correlate with improved mycobacterial control. B cell subsets were characterised among patients with active TB disease (n = 19), LTBI (n = 18) and healthy controls (n = 9) before starting anti-TB treatment. The proportions of mature (**A**), naïve (**B**), classical (**C**), activated (**D**) and atypical (**E**) B cells are shown. Negative correlations between the proportion of activated (**F**) and atypical (**G**) B cells and BCG net growth were seen. Points are single values and bars represent the median with interquartile range. After testing for normality a Kruskal-Wallis test with Dunn’s correction for multiple comparisons was performed. *Represents a p-value of <0.05, **a p-value of <0.005, ***a p-value of <0.0005 and ****a p-value of <0.0001. Spearman’s rho and associated p-values are shown.

There is increasing recognition of the role of B cells (BC) in TB and therefore profiles were characterised in pre-treatment samples (n = 46). Patients with active TB disease and LTBI had a lower proportion of mature BC than healthy controls (median = 16.6%, 12.7%, 19.7%; p = 0.069, p = 0.013, respectively) (Fig. 4A). There was no significant difference in the proportions of naïve BC between the groups (Fig. 4B). Classical memory BC (mBC) were significantly reduced in the active group compared with healthy controls (Fig. 4C). Proportions of activated and atypical mBC were significantly elevated among individuals with active TB and LTBI compared with healthy controls, which negatively correlated with mycobacterial net growth in the MGIT assay (rho = −0.48, p = 0.02; rho = −0.56, p = 0.007, respectively) (Fig. 4D–G).

### IgG1 responses to *M*.*tb*-specific antigens correlate with improved *ex vivo* mycobacterial control

**Figure 5:**
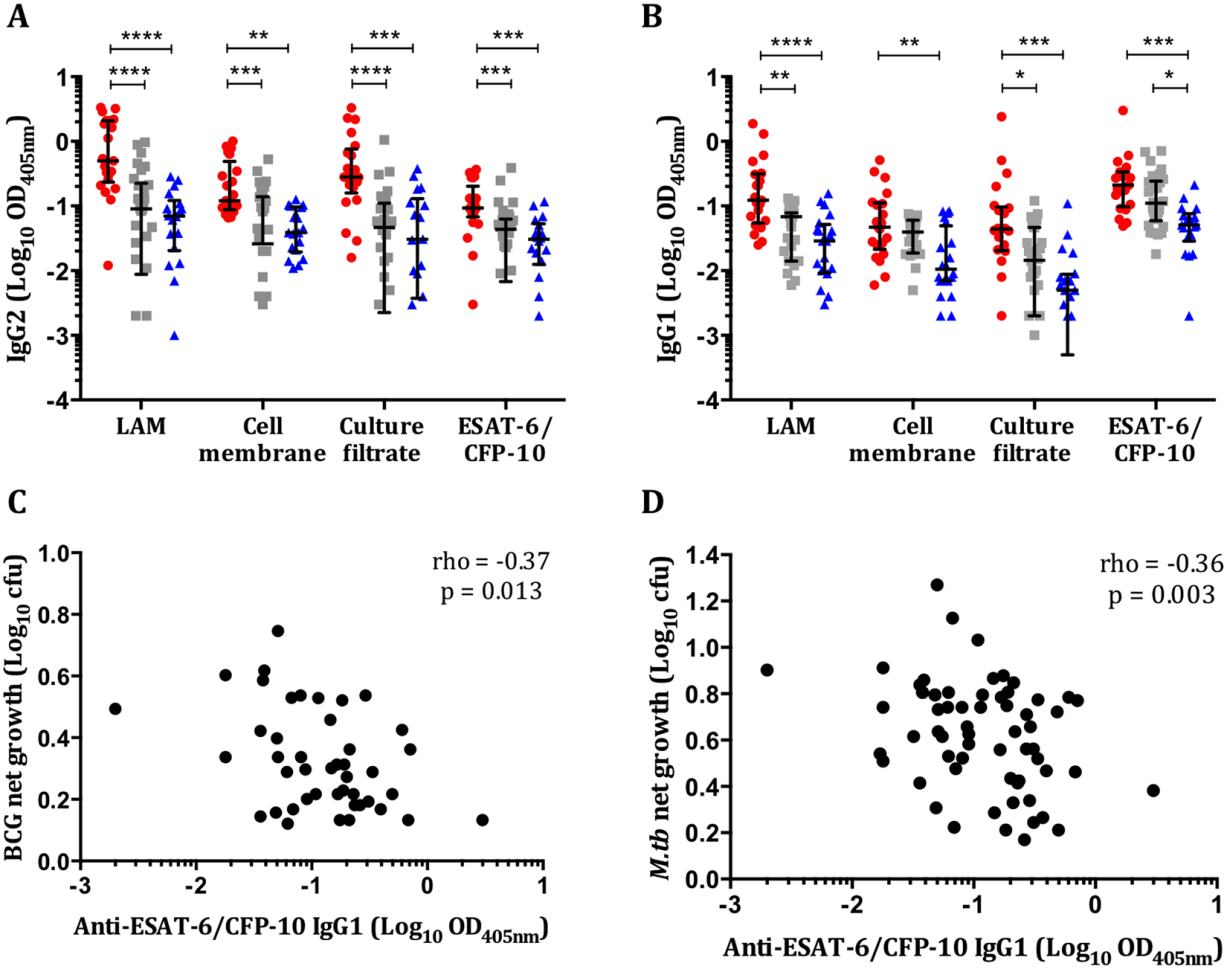
IgG1 responses to RD1 restricted *M*.*tb*-specific antigens are negatively correlated with mycobacterial net growth. Antigen-specific IgG2 (**A**) and IgG1 (**B**) responses in pre-treatment samples from active TB (n = 21), LTBI (n = 30) and healthy control individuals (n = 20) were determined by ELISA. Negative correlations between IgG1 responses to ESAT-6/CFP-10 and BCG (**C**) and *M*.*tb* H37Rv (**D**) net growth were seen. Antigens used were *M*.*tb* H37Rv-derived LAM, cell membrane fraction, culture filtrate and ESAT-6/CFP-10. Optical densities (ODs) are reported following subtraction of the background and plotted on a Log_10_ scale. Points represent the mean of duplicates and bars are the median values with the interquartile range. After normality testing a Kruskal-Wallis test with Dunn’s correction for multiple comparisons was performed for each antigen. *Represents a p-value of <0.05, **a p-value of <0.01, ***a p-value of <0.005 and ****a p-value of <0.0001. Spearman’s rho and associated p-values are shown. Red circles = active TB, grey squares = LTBI and blue triangles = healthy controls.

Serodiagnostics are as yet poorly characterised in the context of *M*.*tb* but is a potentially valuable tool. We examined the distribution of IgG1 and IgG2 responses to protein and non-protein antigens from *M*.*tb* H37Rv in pre-treatment serum samples (n = 71). Significant differences in IgG2 responses between TB versus LTBI, and TB versus controls, were seen against all antigens (Fig. 5A). A similar pattern was seen with IgG1 responses against LAM and culture filtrate antigens (Fig. 5B). The only significant difference in IgG1 responses between LTBI and healthy controls was to ESAT-6/CFP-10 (p = 0.024). Anti-ESAT-6/CFP-10 IgG1 responses were significantly negatively correlated with net growth of both BCG (rho = −0.37, p = 0.013) and *M*.*tb* in the MGIT assay (rho = −0.36, p = 0.003) (Fig. 5C,D). There was a weaker association between anti-culture filtrate IgG1 and *M*.*tb* net growth (rho = −0.057, p = 0.05; data not shown).

### Serum cytokine analysis reveals discrete clusters and strong negative correlations with *ex vivo* mycobacterial growth

**Figure 6:**
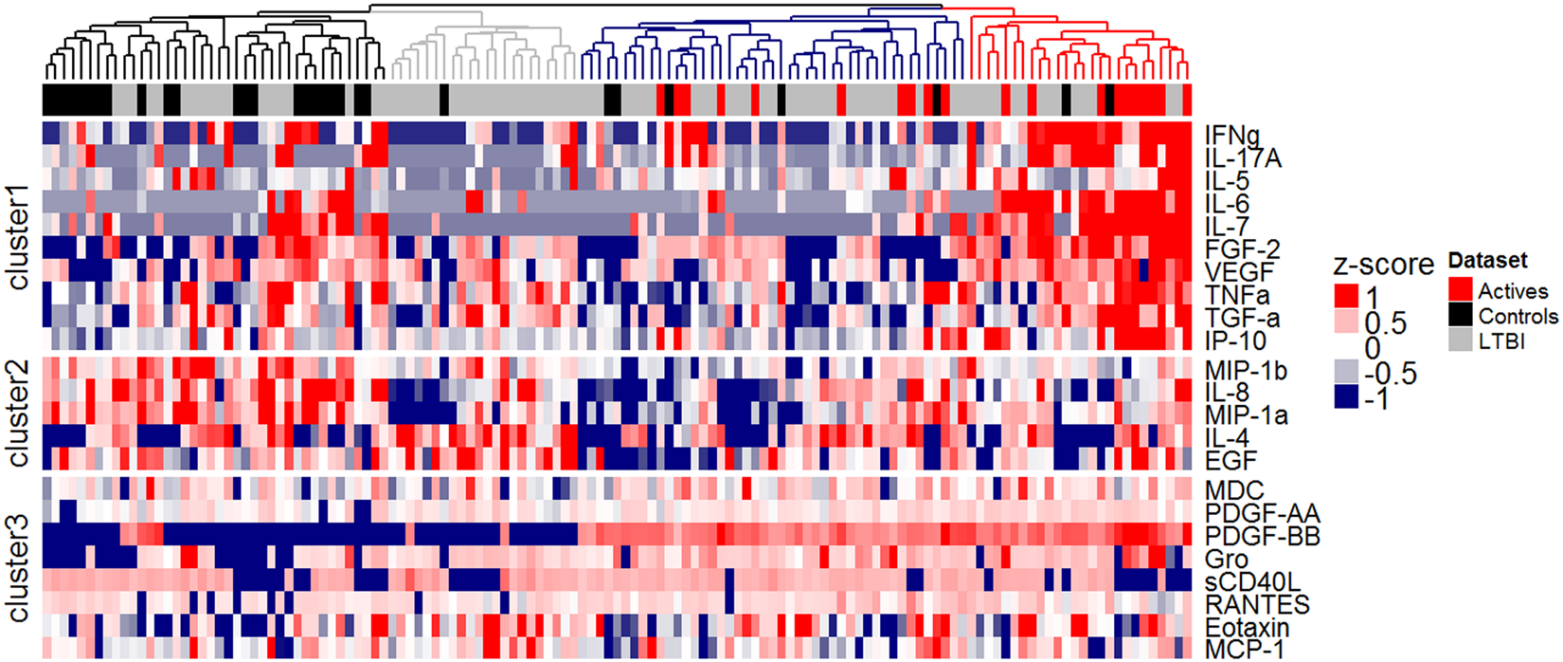
Serum cytokine analysis reveals discrete clusters, which delineate the spectrum of *M*.*tb* infection and strong negative correlations with *ex vivo* mycobacterial growth. Serum cytokine/chemokine responses in pre-treatment samples from active TB (n = 20), LTBI (n = 83) and healthy control individuals (n = 30) were investigated by Luminex assay. A heatmap of cytokine/chemokine levels (log, row scaled, pg/ml) is shown. Samples were clustered according to Spearman correlation distance and the four most distinct clusters were coloured in black, grey, blue and red. For clustering of cytokines/chemokines into three clusters, k-means clustering was applied to the rows.

Differential levels of cytokines/chemokines may indicate immune activation and therefore we measured multiple molecules in pre-treatment serum samples (n = 133). Twenty-three analytes were detected. Clustering analysis identified four homogeneous clusters defining two broad groups of individuals: the red and blue clusters comprising active and some LTBI patients, and the grey and the black clusters of LTBI and control patients (Fig. 6). Specifically, the red cluster contains 10 of the active TB patients, 14 LTBI patients and 2 controls and features very high levels of FGF-2, IFN-γ, IL-5, IL-6, IL-7, IL-17A, IP-10, TGF-α, TNFα and VEGF (cluster 1) and high levels of PDGF-BB, PDGF-AA and Gro (cluster 3). The blue cluster contains the other half of the active patients, 30 LTBI patients and 5 controls, and is characterised by high levels of cluster 3 cytokines/chemokines and low levels of clusters 1 and 2. The black cluster contains 22/30 control patients as well as 18 LTBI patients and cytokine cluster 2 levels are highest here. The grey cluster also features lower cytokine cluster 1 levels and contains 21/83 LTBI patients and one control patient. Spearman correlation coefficients showed significant negative associations between *M*.*tb* net growth and serum concentrations of Gro, TGF-α, PDGF-BB, PDGF-AA, IP-10, and MDC (Supplementary Table 2).

## Discussion

Identifying individuals with LTBI at risk of developing active disease as candidates for preventative treatment will be crucial to control TB, and fundamental to achieving this is to understand the full array of infection. We present evidence supporting the concept of a heterogenous spectrum of *M*.*tb* infection, potentially an important initial step in stratifying reactivation risk in LTBI. Using a functional MGIA we developed a continuous ordinal scale to define *in vivo M*.*tb* infection status, based on an individual’s *ex vivo* ability to control mycobacterial growth. The simple ordinal readout of anti-mycobacterial immunity reflects the summative combination of multiple individual cellular and non-cellular constituents. The MGIA assay discriminated between individuals with different states of *M*.*tb* infection, with control of mycobacterial growth greatest in those with active TB and least in healthy controls. We hypothesise that the improved control seen in this model may reflect a general state of immune activation, generated in response to high *in vivo* bacillary loads. This may also account for the wide spectrum of mycobacterial growth control seen among participants with LTBI, with many overlapping with the active and control groups. We suggest that some individuals with a high bacillary burden but subclinical disease demonstrate enhanced immune activation and thus bacillary control similar to the active group, whilst those with quiescent infection cluster with the healthy controls. This is supported by human intradermal BCG vaccination studies, which have shown induction of immune activation and improved growth control in MGIAs^10,11^. Such a principle is well established in animal models of infection, where prior activation macrophages has been shown to enhance control of *Salmonella typhimurium*^12^.

The significant increases in mycobacterial net growth observed after treatment, which were of greatest magnitude among the active disease patients, are consistent with our hypothesis. Individuals with the highest bacillary burden, and the greatest immune activation, demonstrated larger changes in growth control after therapy, supported by resolution of pre-treatment hematological abnormalities.

The differences in the discriminatory power of the MGIT assay using either BCG or *M*.*tb* may be due to mycobacterial virulence. While significant differences in growth control occurred between all groups when using BCG, the *M*.*tb* assay was unable to differentiate between active and LTBI groups. We have hypothesised that differential mycobacterial control results from immune activation and suggest that the increased virulence of *M*.*tb* H37Rv (as demonstrated by greater growth rates) may have overcome such activation in the active TB group, thus reducing the ability to distinguish between individuals with active TB or LTBI groups. This observation has important implications for further work to assess infection stratification using this MGIA.

The heterogeneous responses in the *M*.*tb* assay among individuals with active TB may reflect clinical diversity. While this heterogeneity did not appear to be associated with different disease states (e.g extrapulmonary versus pulmonary TB), this may be due the relatively small extrapulmonary TB sample size in the current study.

The increased ML ratio, neutrophil and platelet counts, and reduced hemoglobin concentrations seen here before treatment among individuals with active disease, and some with LTBI, are consistent with other reports^13–17^. Such profiles may result in altered immune-modulatory and anti-mycobacterial functions, resulting in enhanced *ex vivo* control. Bacillary growth correlated with hemoglobin concentration and iron is known to augment bacillary replication in MGIA models^18,19^. The negative association between ML ratio and mycobacterial growth may reflect the balance between innate and adaptive responses in the assay and could prove a useful marker of mycobacterial disease risk^14,20^. Interestingly, after treatment the correlation between mycobacterial growth and ML ratio reversed, resulting in profiles very similar to those reported among healthy individuals, suggesting that treatment alters not only the absolute number of myeloid and lymphoid cells, but also their function^21^.

We used an exploratory multi-platform approach to further characterise the spectrum of mycobacterial control profiles defined by the MGIA. We observed increased frequencies of intermediate monocytes and decreases in non-classical monocytes among individuals with active disease and LTBI, which changed significantly in the active group following anti-TB treatment, findings consistent with other reports^22,23^. There is biological plausibility for the significant correlations between pre-treatment intermediate (negative) and non-classical (positive) monocytes and MGIA bacillary growth. Intermediate monocytes typically account for up to 5% of total monocytes, are pro-inflammatory and produce high levels of TNF-α, IL-1β and IL-6^24,25^. Non-classical (‘patrolling’) monocytes represent 5–10% of circulating monocytes and survey the endothelium for signs of inflammation or damage, which induces transmigration and the production of TNF-α and IL-1β^25^. The sequestration of non-classical monocytes in active disease would account for the reduction in peripheral frequencies, while enhanced numbers of inflammatory intermediate monocytes *in vivo* may result in the improved *ex vivo* mycobacterial control.

While there is increasing evidence of B cell responses to intracellular pathogens, little is known about B cell subsets in TB^26,27^. We found that individuals with active TB had significant increases in activated and atypical mBCs and significantly fewer classical mBC. We observed strong negative correlations between the proportion of atypical or activated mBCs and *ex vivo* mycobacterial growth. Atypical mBCs are hypo-responsive cells which are thought to be generated from dysfunctional germinal centers or pre-existing classical mBCs^28,29^. The resulting diminished classical mBC frequencies are in agreement with our observations. Increased proportions of atypical mBCs occur in active TB and LTBI, and these cells have impaired capacity for proliferation, immunoglobulin and cytokine production, which resolves following treatment^30^. In TB, expanded atypical mBCs and failure of B cell responses could contribute to reactivation of LTBI, akin to the reduced malaria immunity observed following repeated *Plasmodium falciparum* infections, which result in expanded atypical mBC populations^31^.

The ability to discriminate between active TB and LTBI or controls by IgG1 or IgG2 responses is consistent with evidence that antibody titers are related to mycobacterial load and disease severity^32,33^. One of the difficulties with TB serodiagnostics is the large overlap in responses, particularly between uninfected healthy controls and LTBI^34^. Prior environmental mycobacteria exposure and BCG vaccination could account for high IgG background levels. However, IgG1 responses to the RD1-restricted protein antigens ESAT-6/CFP-10 did discriminate between LTBI and healthy controls. The strong negative association of these responses with *ex vivo* mycobacterial growth suggests that this *M*.*tb*-specific antigen may reflect bacillary burden and therefore disease status. ESAT-6 and CFP-10 are mainly secreted during bacterial replication but there is evidence for ongoing bacillary replication in LTBI^35^. Anti-ESAT-6/CFP-10 IgG1 responses may correlate with mycobacterial activity, in contrast to the other antigens which are likely to be present irrespective of the replicative state of mycobacteria.

Finally, our analysis of serum cytokines/chemokines supports the hypothesis that the magnitude of *ex vivo* inhibition of bacillary growth is related to the degree of *in vivo* immune activation^36^. Hierarchical cluster analysis highlighted several correlations between high analyte levels and enhanced mycobacterial control. Among these, IP-10 and PDGF- BB are particularly noteworthy. The chemokine IP-10 (CXCL10) is involved in the regulation of innate and adaptive immune responses through the recruitment of monocytes and activated T-cells to sites of inflammation^37^. High levels of IP-10 have been reported in active TB disease, possibly as a non-specific indication of inflammation and immune activation, thus reflecting disease activity^38,39^. PDGF-BB, an angiogenic growth factor, was also negatively associated with *ex vivo* mycobacterial growth. Recent studies have shown increased serum levels of PDGF-BB in patients with pulmonary TB, which was associated with pulmonary fibrosis and reduced following treatment^40,41^. Serum PDGF-BB may reflect the number, size and/or composition of pulmonary TB granulomas and therefore the underlying state/degree of infection.

There were some limitations to this study. Peripheral blood samples are unlikely to simulate the complexity of the pulmonary granuloma in TB, however they are easily accessible and are frequently used in diagnostics, allowing comparison between studies. In addition, while we consider our findings biologically plausible, they are correlative observations. We did not have the opportunity to investigate underlying mechanisms, which will be an area for future studies. Finally, we acknowledge that this exploratory work was not a biomarker study and suggest that further studies in independent cohorts are required to validate the MGIA findings and associated immune responses presented here.

**Figure 7:**
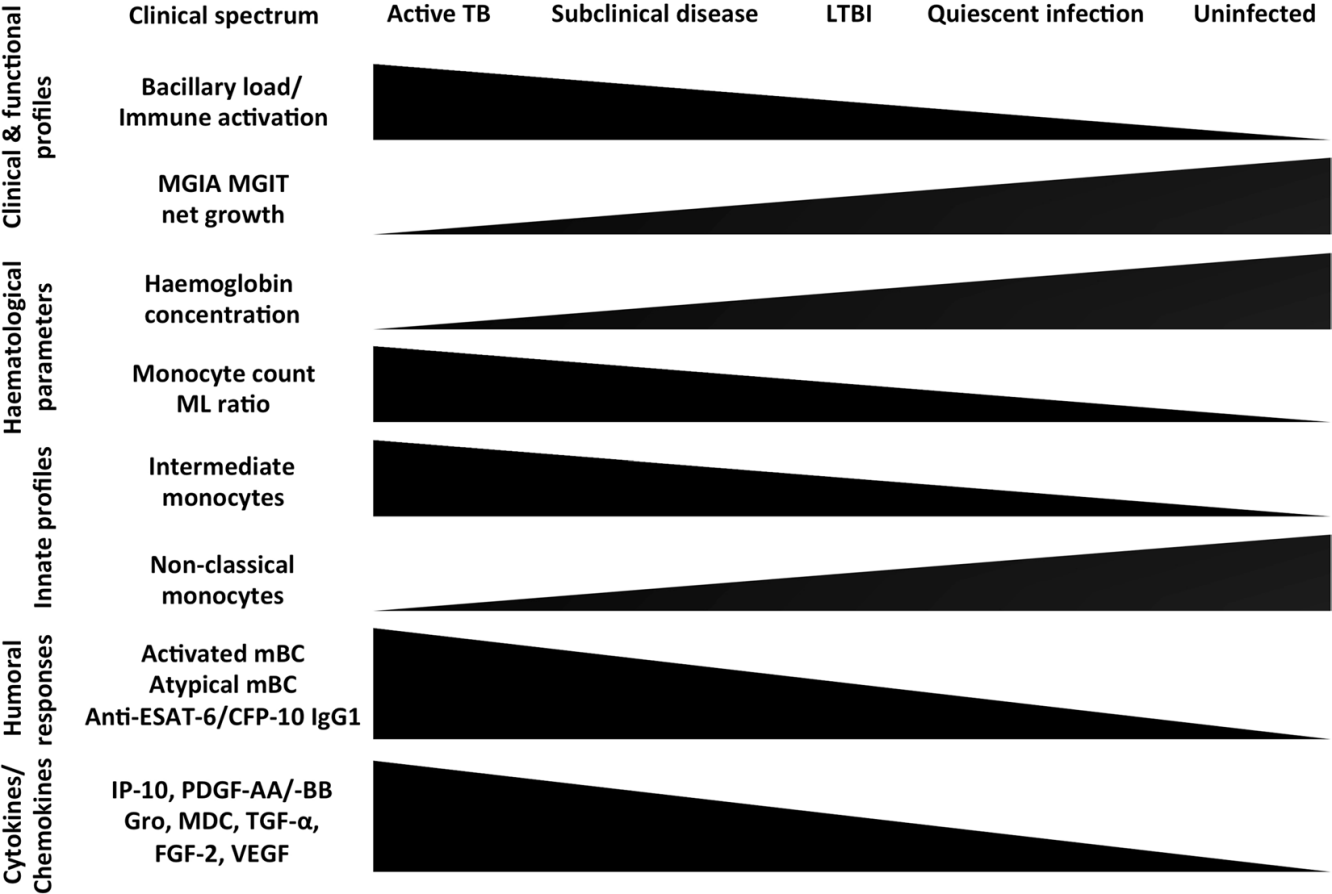
A spectrum of *M*.*tb* infection and associated immune profiles. We propose that the delineation of the spectrum of *M*.*tb* infection identified by the MGIT MGIA, and the underlying immunological profiles associated with this spectrum, is biologically plausible and may have value in identifying subclinical active TB disease and possibly in determining reactivation risk of LTBI.

Using a novel approach we have described the spectrum of TB using a functional MGIA and further characterised immune profiles associated with mycobacterial control responses (Fig. 7). Our findings may have value in identifying subclinical active TB disease and possibly in determining reactivation risk.

## Methods

### Clinical samples

Studies using samples collected from human volunteers were conducted in accordance with the principles of the Declaration of Helsinki, Good Clinical Practice, and local ethical and regulatory requirements. All volunteers provided informed consent for study participation. Peripheral blood samples were collected from HIV-negative adult volunteers (≥18 years) with either a suspected (based on clinical suspicion and radiological/histological evidence) or microbiologically confirmed new diagnosis of pulmonary or extrapulmonary TB disease, or with a new diagnosis of LTBI (a positive IFN-γ release assay (IGRA)), in the absence of any other significant co-morbidity. Participants were enrolled at one of three clinical sites in Birmingham, Oxford or Catterick, UK, under ethical approval granted by the *National Research Ethics Service*, Heart of England NHS Foundation Trust (2012107RM), Committee South Central - Oxford C (12/SC/0299) and the Ministry of Defence Research Ethics Committee (MoDREC 237/PPE/11), respectively. In addition, samples were also collected from healthy uninfected volunteers (with no past medical history of TB, exposure to *M*.*tb* or evidence of LTBI) from these sites and also at the Jenner Institute, Oxford, in accordance with Oxford University policy.

### Bacterial strains, culture and titrations

All experimental work using *M*.*tb* was performed in an approved containment level 3 (CL3) laboratory. Frozen 1 ml aliquots of strain stocks were thawed and grown in 100 ml Middlebrook 7H9 media with 10% OADC to mid-log phase then divided into 1 ml aliquots and stored at −80 °C until required. Mycobacterial stocks were titrated by serially diluting in supplemented 7H9 media and culturing in BACTEC MGIT tubes supplemented with PANTA antibiotics and OADC enrichment broth (Becton Dickinson, UK), as previously described^42^. In addition, the number of colony forming units (cfu) for each dilution was determined by solid culture on 7H10 agar plates. A standard curve of time-to-positivity (TTP) against cfu was derived and linear regression analysis generated an equation to convert experimental TTP to cfu.

### *Ex vivo* whole blood MGIA

This assay was performed as previously described^19^. Duplicate tubes containing 300 μl of whole blood were incubated with 300 μl of RPMI (containing 10% pooled human serum, 2mM L-glutamine and 25 mM HEPES; Sigma, UK) inoculated with ~150 cfu BCG or *M*.*tb* on a 360° rotator at 37 °C for 96 hours (volume of the mycobacterial stock was calculated to give a TTP of 6.5 days, previously determined to give optimal differential responses). Cells were then lysed with sterile water, the mycobacteria resuspended in 7H9 media and transferred to a BACTEC MGIT tube supplemented with PANTA antibiotics and OADC enrichment broth (Becton Dickinson, UK). Tubes were placed in the BACTEC 960 machine and incubated at 37 °C until the detection of positivity by fluorescence (TTP). In addition, on day 0, duplicate viability control tubes were set up by directly inoculating supplemented BACTEC MGIT tubes with the same volume of mycobacteria as the samples. The mean TTP for duplicates was converted to a cfu count (as described above) and net growth ratio was calculated as Log_10_(sample cfu/control cfu). A smaller net growth value indicates less bacillary replication and therefore represents greater mycobacterial control. Samples failing to meet pre-defined reproducibility criteria of ΔTTP < 6 hours between duplicates, as determined by initial experiments, were excluded.

### Peripheral blood mononuclear cell isolation, cryopreservation and thawing

Peripheral blood mononuclear cells (PBMC) were separated from fresh heparinized whole blood obtained from study participants by density gradient centrifugation. Briefly, 15–30 ml of whole blood was carefully layered onto the membrane a Leucosep tube (Greiner Bio-One) pre-loaded with LymphoPrep**™** (StemCell Technologies, France) and centrifuged. PBMC were collected from the interface and washed twice with pre-warmed R0 media (RPMI 1640, 2mM L-glutamine, 100U/ml penicillin and streptomycin and 1% sodium pyruvate; Sigma Aldrich, UK), then resuspended in R10 media (R0 media with 10% fetal calf serum (FCS); Sigma Aldrich) and counted using a CASY^®^ counter (Roche, Switzerland). For cryopreservation, cells were centrifuged and resuspended in ice-cold FCS to which an equal volume of 20% DMSO in FCS was added to give a concentration of 5–10 × 10^6^ cells/ml, aliquoted into cryovials and placed in a CoolCell^**®**^ cell freezing container which was stored at −80 °C until transfer to liquid nitrogen after 1–2 days. Cryopreserved PBMC were rapidly thawed in a 37 °C waterbath, transferred to a tube containing pre-warmed R10, centrifuged and resuspended in R10 containing 2 μl/ml of benzonase (Merck Chemicals) and rested at 37 °C, 5% CO_2_, for a minimum of 2 hours before counting and use in subsequent assays.

### Immunophenotyping monocytes and B cells

Thawed PBMC were counted and diluted to a final concentration of 5 × 10^6^/ml in R10 media. After washing, cells (1 × 10^6^) were stained with Aqua viability dye (Invitrogen, USA) before staining with fluorochrome-conjugated antibodies to surface markers. For monocyte immunophenotyping, the surface panel included anti-CD3 AF700 (eBioscience), anti-CD19 Pacific Blue (Life Technologies), anti-CD56 Pacific Blue, anti-HLA-DR APC-Cy7, anti-CD14 PE and anti-CD16 PE-Cy7 (BioLegend). The B cell (BC) surface panel contained anti-CD10 APC and anti-CD19 PECy7 (Becton Dickinson), anti-CD21 Pacific Blue (eBioscience) and anti-CD27 Qdot605 (Invitrogen). Cells were stained in batches with either the B cell or monocyte panel, washed with FACS buffer (BD Pharmingen) and fixed with 1% paraformaldehyde (Sigma, UK). Fluorescence-minus-one (FMO) controls were included in each batch. Compensation was performed using CompBeads (BD Biosciences) stained with each fluorophore and compensation matrices were calculated with FACSdiva. Stained cells were acquired using an LSRII flow cytometer (BD Biosciences) within 24 hours and analyzed using FlowJo v8.3 (Tree Star Inc., USA). Gating strategies and an example of raw flow cytometry data are shown in Supplementary Figs 2 and 3. Monocyte subsets were defined as CD14++CD16− (classical), CD14++CD16+ (intermediate), or CD14+CD16+ (non-classical), as previously described^43^. B cell subsets were defined as CD19+CD10− (mature B cells), CD27+CD21− (activated memory B cells, mBC), CD27+CD21+ (classical mBC), CD27−CD21+ (naïve mBC) and CD27−CD21− (atypical mBC), as previously described^44^.

### Anti-*M*.*tb* IgG isotype enzyme-linked immunosorbent assays

Microtiter plates were coated overnight at room temperature with the following antigens (at final concentrations) prepared from *M*.*tb* H37Rv (BEI Resources): cell membrane fraction (2 μg/ml), culture filtrate (2 μg/ml), LAM (2 μg/ml) and a 1:1 mix of ESAT-6/CFP-10 (10 μg/ml each). Each plate was prepared in duplicate to test IgG1 and IgG2 responses, and also included native human IgG1 and IgG2 (2 μg/ml; AbD Serotec, UK) as positive controls and PBS as a negative control. After washing with PBS containing 0.05%/Tween 20 (PBS/T) and blocking with casein, serum samples were tested in duplicate at 1:100 (diluted in casein) for all antigens except for ESAT-6/CFP-10 (1:10 dilution). Casein was used for control wells. After incubation at room temperature, biotinylated secondary antibody (anti-IgG1 or anti-IgG2; Life Technologies) at 1:1000 dilution was added, followed by washing and the addition of Extravidin-ALP (1:5000; Sigma Aldrich). pNPP substrate (Sigma Aldrich) was added and plates were read at 405 nm every 10 minutes using an ELx800 Microplate Reader with Gen5 software until the IgG1 and IgG2 isotype controls reached an optical density (OD) of 0.6 and 1.2, respectively (previously determined from the gradient of a standard curve of native human IgG1 or IgG2 ranging from 0.1 to 100 μg/ml to avoid saturation). The mean OD of negative controls was subtracted from the mean OD of samples and readings with an OD > negative control plus 3 standard deviations (SD) were considered positive.

### Multiplex serum cytokine and chemokine analysis

The Milliplex^**®**^ Map human cytokine/chemokine system (Merck Millipore), based on a luminex bead array platform, was used to measure the concentrations of 41 analytes. Levels of interleukin (IL)-1α, IL-1β, IL-1ra, IL-2, IL-3, IL-4, IL-5, IL-6, IL-7, IL-8, IL-9, IL-10, IL-12 (p40), IL-12 (p70), IL-13, IL-15, IL-17A, inducible protein (IP)-10, fibroblast growth factor (FGF)-2, eotaxin, fractalkine, granulocyte colony stimulating factor (G-CSF), granulocyte macrophage colony stimulating factor (GM-CSF), macrophage inflammatory protein (MIP)-1α, MIP-1β, Flt-3 ligand, platelet derived growth factor (PDGF)-AA, PDGF-BB, tumour growth factor (TGF)-α, tumour necrosis factor (TNF)-α, TNF-β, vascular endothelial growth factor (VEGF), monocyte chemoattractant protein (MCP)-1, MCP-3, interferon (IFN)-α2, IFN-γ, macrophage-dervied chemokine (MDC), epidermal growth factor (EGF), soluble CD40 ligand (sCD40L), GRO and regulated on activation, normal T cell expressed and secreted (RANTES) were measured in unstimulated serum samples, according to the manufacturer’s instructions. Plates were read using the Biorad Luminex^**®**^100**™** system and analyzed with integrated Bioplex Manager Software v6.1 (Biorad Corp., USA). Values above or below the concentration range for each analyte (3.2 to 10,000 pg/ml) were derived from analyte-specific standard curves by extrapolation. Below the lowest extrapolated value a concentration of zero was recorded.

### Statistical analysis

Analyses used Prism (v6.0) and SPSS (v22). The D’Agnostino-Pearson omnibus test was used to assess normality of data. Mean values of parametric data were compared between groups using paired t-tests, unpaired t-tests, repeated measures and one-way ANOVAs, with Dunnett’s post-test correction for multiple comparisons. Median values of non-parametric data were compared between groups using Wilcoxon matched-pairs signed rank tests, Mann-Whitney tests, and Kruskal-Wallis tests, with Dunn’s post-test correction. For correlation analyses, Pearson’s or Spearman’s tests were performed after testing for normality. Reported P values are two-sided and a value of less than 0.05 was considered significant. For hierarchical clustering, luminex data were log transformed, mean centered and scaled. One minus Spearman correlation coefficients were then used as dissimilarity metrics to cluster samples, and average linkage as the agglomeration rule was used as implemented in the hclust command in R. Clusters were then coloured according to branch height and the number of most informative clusters was estimated to be four by the method described in as implemented in the package NbClust^45,46^. The final Heatmap was assembled using the ComplexHeatmap package in R^47^.

## Electronic supplementary material

Supplementary Information

## Data Availability

All data generated or analysed during this study are included in this published article (and its Supplementary Information files).
