# Supplementary material for: Immunological correlates of mycobacterial growth inhibition describe a spectrum of tuberculosis infection
Source: Sci Rep. 2018 Sep 27;8:14480. doi: 10.1038/s41598-018-32755-x (PMC6160428; doi:10.1038/s41598-018-32755-x)
Supplement: Supplementary file 1 — Supplementary Information [file 41598_2018_32755_MOESM1_ESM.pdf]

Supplementary Information for:

Immunological correlates of mycobacterial growth inhibition describe a spectrum of tuberculosis infection

Authors

Matthew K. O'Shea[1,2*], Rachel Tanner[1], Julius Müller[1], Stephanie A. Harris[1], Danny Wright[1], Lisa Stockdale[3], Elena Stylianou[1], Iman Satti[1], Steven G. Smith[3], James Dunbar[4], Thomas E. Fletcher[5], Martin Dedicoat[6], Adam F. Cunningham[7], Helen McShane[1].

Author affiliations

[1]The Jenner Institute, Nuffield Department of Medicine, University of Oxford, Oxford, UK.

[2]Institute of Microbiology and Infection, University of Birmingham, Birmingham, UK.

[3]Department of Immunology and Infection, London School of Hygiene and Tropical Medicine, London, UK.

[4]The Friarage Hospital, Northallerton, Yorkshire, UK.

[5]Liverpool School of Tropical Medicine and Hygiene, Liverpool, UK.

[6]Heart of England Foundation Trust, Birmingham, Birmingham, UK.

[7]Institute of Immunology and Immunotherapy, University of Birmingham, Birmingham, UK.

Supplementary Table 1. Pre- and post-treatment hematology parameters of study participants.

Laboratory parameter Median [IQR]	Pre-treatment						Post-treatment			
	State of infection			P values			State of infection		P values	
	Active TB n=19	LTBI n=129	Controls n=35	P1	P2	P3	Active TB n=11	LTBI n=78	P4	P5
**Total leucocytes ($x10^9$/L)**	7.38 [5.85-9.03]	6.80 [5.64-8.20]	7.30 [5.80-8.40]	ns	ns	ns	5.62 [4.63-6.98]	5.37 [4.80-6.57]	*	****
**Neutrophils ($x10^9$/L)**	5.28 [2.99-7.24]	4.1 [3.01-5.10]	4.3 [3.34-5.20]	ns	ns	ns	2.64 [2.11-4.02]	3.00 [2.50-3.65]	**	****
**Lymphocytes ($x10^9$/L)**	1.46 [1.28-1.87]	1.90 [1.50-2.36]	2.00 [1.70-2.40]	*	**	ns	1.93 [1.57-2.26]	1.70 [1.42-2.16]	ns	**
**Monocytes ($x10^9$/L)**	0.63 [0.57-0.92]	0.42 [0.37-0.50]	0.40 [0.32-0.50]	****	****	ns	0.46 [0.40-0.61]	0.40 [0.30-0.50]	**	*
**ML ratio**	0.49 [0.32-0.78]	0.24 [0.19-0.29]	0.19 [0.17-0.28]	****	****	ns	0.21 [0.16-0.28]	0.23 [0.18-0.29]	**	ns
**Eosinophils ($x10^9$/L)**	0.16 [0.07-0.3]	0.17 [0.1-0.27]	0.2 [0.1-0.23]	ns	ns	ns	0.18 [0.14-0.30]	0.10 [0.10-0.20]	ns	****
**Basophils ($x10^9$/L)**	0.04 [0.02-0.07]	0.04 [0.0-0.1]	0.0 [0.0-0.1]	ns	ns	ns	0.04 [0.03-0.05]	0.0 [0.0-0.06]	ns	****
**Haemoglobin (g/L)**	135.0 [119.0-150.3	148.0 [139.0-155.5]	154.0 [147.0-158.0]	*	***	*	159.0 [139.0-166.0]	147.5 [138.0-155.5]	**	*
**Mean cell volume (fL)**	85.9 [83.1-88.5]	89.1 [84.9-92.5]	88.3 [85.8-90.6]	ns	ns	ns	87.8 [84.5-88.7]	89.9 [86.6-92.9]	ns	ns
**Platelets ($x10^9$/L)**	324.5 [210.0-521.5]	233.5 [204.0-272.5]	243.0 [212.8-270.8]	*	ns	ns	250.0 [208.0-326.0]	227.0 [194.5-249.5]	*	****

After testing for normality, P values for pre-treatment haematology parameters were calculated using a Kruskal-Wallis test with Dunn's correction for multiple comparisons between infection states as follows: P1=Active TB vs LTBI; P2=Active TB vs Healthy controls; P3=LTBI vs Healthy controls.

P values were subsequently calculated using a Wilcoxon matched-pairs signed rank test between pre- and post-treatment haematology parameters as follows: P4=Active TB pre- vs post-treatment; P5=LTBI pre- vs post-treatment.

* represents a p-value of <0.05, ** a p-value of <0.005, *** a p-value of <0.0005 and **** a p-value of <0.0001.

LTBI=latent TB infection; IQR=interquartile range; ns=non-significant; ML=monocyte-to-lymphocyte.

Supplementary Table 2. Correlation between MGIT *M.tb* net growth and serum cytokine/chemokine concentrations (Spearman's rho correlation coefficient).

Cytokine/chemokine	rho	P value
Gro	-0.316	2.32E-04
TGF-α	-0.286	9.22E-04
PDGF-BB	-0.257	3.02E-03
PDGF-AA	-0.242	5.27E-03
IP-10	-0.220	1.17E-02
MDC	-0.184	3.58E-02
MIP-1b	0.163	6.33E-02
MIP-1a	0.152	8.24E-02
FGF-2	-0.144	1.02E-01
IL-4	-0.129	1.43E-01
VEGF	-0.099	2.62E-01
IL-8	0.093	2.89E-01
sCD40L	0.084	3.37E-01
IL-6	-0.084	3.41E-01
IL-17A	-0.064	4.65E-01
EGF	0.059	5.01E-01
MCP-1	0.057	5.15E-01
Eotaxin	-0.055	5.34E-01
IFN-γ	-0.045	6.12E-01
IL-5	-0.035	6.89E-01
RANTES	0.007	9.41E-01
IL-7	0.006	9.49E-01
TNF-α	-0.004	9.61E-01

Supplementary Figure 1.

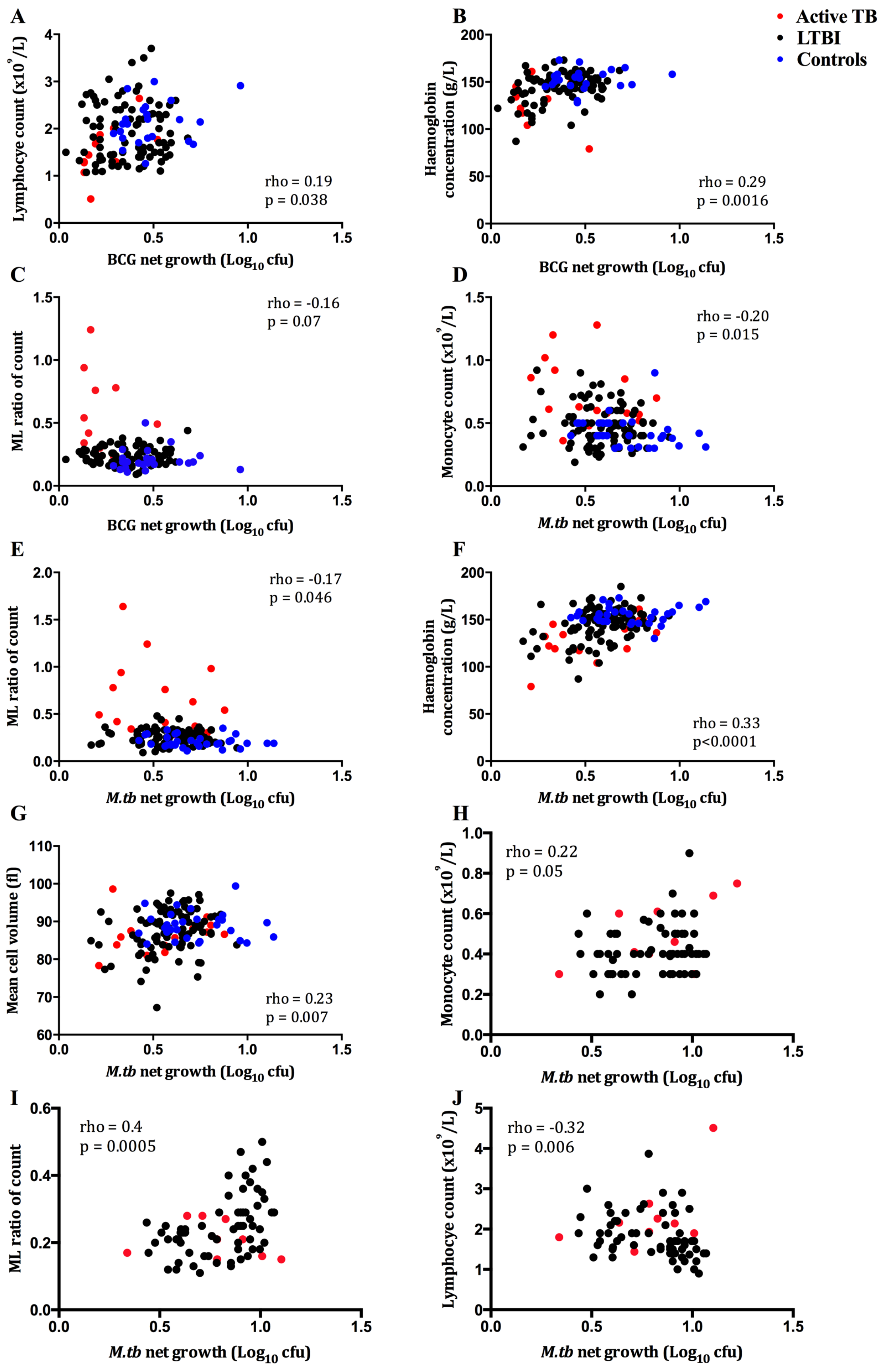

Supplementary Figure 1. ***Ex vivo*** **mycobacterial control correlates with** ***in vitro*** **haematological markers.** The relationship between baseline and post-treatment mycobacterial net growth and haematology parameters was investigated. After testing for normality, Spearman's correlations were calculated between pre-treatment haematology data and the whole blood BCG (3A-C) and *M.tb* H37Rv (3D-G) MGIT results. Associations were found between BCG net growth and lymphocyte count (A), haemoglobin concentration (B) and monocyte-to-lymphocyte (ML) ratio (C). Significant correlations were also found between *M.tb* H37Rv net growth and monocyte count (D), ML ratio (E), haemoglobin concentration (F) and mean cell volume (G). Spearman's correlations showed significant associations between post-treatment *M.tb* H37Rv net growth and monocyte count (H), monocyte-to-lymphocyte (ML) ratio (I), and lymphocyte count (J). Red circles = active TB, black = LTBI and blue = healthy controls.

Supplementary Figure 2.

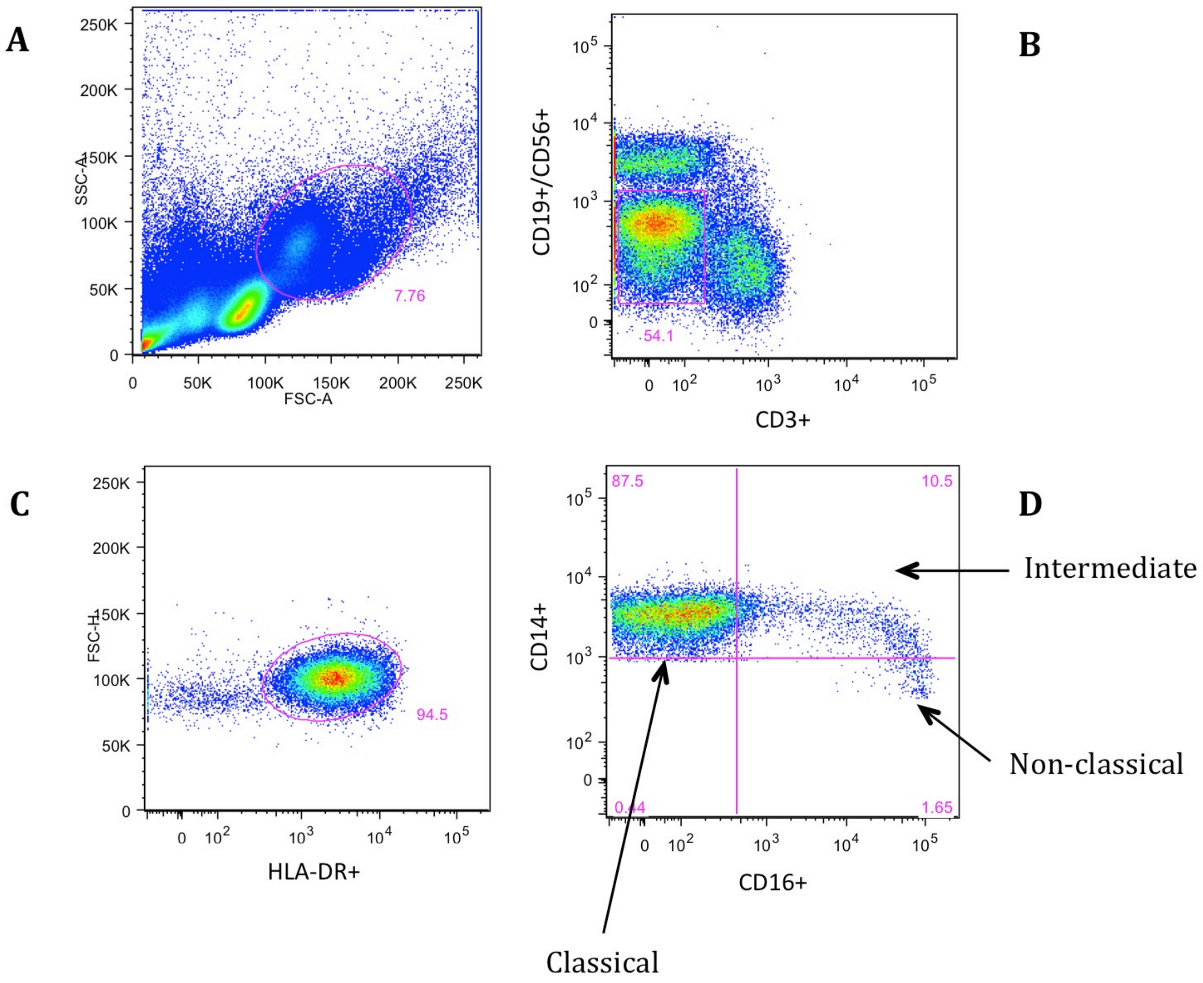

Supplementary Figure 2. Monocyte gating strategy. Cells were visualized by size (SSC-A vs FSC-A) and a large gate was drawn around the monocyte cloud, excluding most cell debris (A). After selecting live cells, CD3+, CD19+ and CD56+ cells were all excluded (B). From the double negative cells, HLA-DR+ cells were then selected (C) and were discriminated on a bivariate scatter plot of CD14+ vs CD16+ (D). Double negative cells on this plot were removed as non-monocytes. Monocyte subsets were defined as CD14++CD16- (classical), CD14++CD16+ (intermediate), or CD14+CD16+ (non-classical) on the basis of FMO gating [3]. The proportion of each subset of the total monocyte population was calculated.

Supplementary Figure 3.

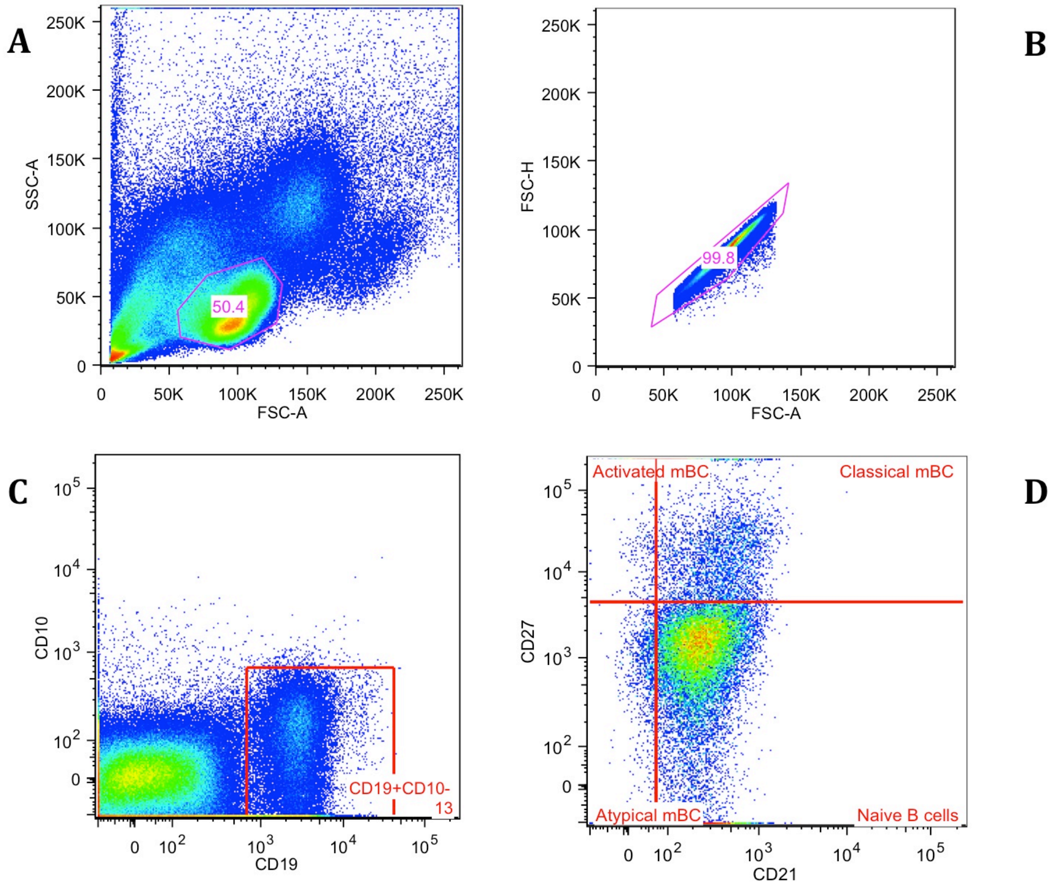

Supplementary Figure 3. B cell gating strategy. Cells were visualized by size (SSC-A vs FSC-A) and a large gate was drawn around the lymphocyte cloud, excluding most cell debris (A). Singlets and live cells were then selected (B). After selecting for mature B cells (CD19+CD10-) (C), B cells subsets were discriminated on a bivariate scatter plot of CD27+ vs CD21+ (D). B cells were defined CD27+CD21- (activated mBC), CD27+CD21+ (classical mBC), CD27-CD21+ (naïve mBC) and CD27-CD21- (atypical mBC) on the basis of FMO gating [4]. Mature B cells were calculated from the total lymphocyte population and other subsets as a proportion of mature B cells.
